# Neurovascular coupling and cerebrovascular reactivity: physiology, lifespan development, pathophysiology, and clinical relevance

**DOI:** 10.3389/fphys.2026.1728065

**Published:** 2026-03-03

**Authors:** Zena Khartabil, David Kala, Yeva Prysiazhniuk, Jakub Otáhal

**Affiliations:** 1 Department of Pathophysiology, Second Faculty of Medicine, Charles University, Prague, Czechia; 2 Epilepsy Research Center Prague, EpiReC, Prague, Czechia

**Keywords:** arterial spin labelling, cerebral autoregulation, cerebral perfusion, cerebrovascular reactivity, functional magnetic resonance imaging, hypercapnic challenge, neurovascular coupling, transcranial doppler

## Abstract

Neurovascular coupling (NVC) and cerebrovascular reactivity (CVR) are two key regulatory mechanisms that maintain adequate cerebral blood flow (CBF) to meet the metabolic demands of the human brain. In this review, we summarize the essential physiological principles needed to understand the distinctions, similarities, and overlaps between these systems, providing a deeper comprehension of cerebral perfusion and its regulation. We further examine their development across the lifespan, including embryogenesis and early life, with a focus on how synaptogenesis, myelination, and synaptic pruning shape NVC, CVR, and CBF. Finally, we discuss the effects of aging, emphasizing cerebrovascular remodeling and its consequences. Various neuropathologies are then explored from the perspective of altered CBF regulation, highlighting numerous correlations between regulatory dysfunction and disease pathogenesis. We also review a range of investigative techniques used to assess NVC and CVR, including (but not limited to) arterial spin labelling, functional magnetic resonance imaging, and transcranial Doppler. This review systematically integrates the developmental trajectories of NVC and CVR across the human lifespan with their pathophysiological alterations and clinical assessment methods. By combining developmental, mechanistic, and clinical perspectives, it provides a comprehensive framework that highlights how age-related changes shape cerebrovascular regulation, with important implications for both research and the interpretation of neurovascular data.

## 1 Introduction

The human brain accounts for only 2% of total body weight, yet it demands more than 20% of the body’s oxygen and nutrient supply (Takahashi, 2022). A meticulous interplay of regulatory mechanisms maintains cerebral blood flow (CBF) to match neural metabolic activity. Neurovascular coupling (NVC) and cerebrovascular reactivity (CVR) are two key components of this regulation. Although these terms are sometimes used interchangeably in the literature, they represent distinct but partly overlapping processes.

This review defines and differentiates NVC and CVR, outlines their molecular and cellular mechanisms, and places them within a developmental lifespan framework - from embryogenesis and postnatal maturation to adulthood and aging. Previous reviews have focused mainly on individual molecular pathways, single disease entities, or specific imaging approaches. A comprehensive synthesis integrating developmental changes, physiological mechanisms, disease-related alterations, and clinical assessment methods has not yet been undertaken.

The aim of this review is threefold: (i) to delineate the molecular and cellular basis of NVC and CVR and their vascular anatomical correlates; (ii) to contextualize these mechanisms within maturation and remodeling across the human lifespan, including key developmental processes such as synaptogenesis, myelination, synaptic pruning, and age-related vascular changes; and (iii) to link these processes to major disease groups and their diagnostic evaluation in experimental and clinical research.

## 2 Cerebral blood flow

### 2.1 Physiology of CBF

Cerebral blood flow is defined as the volume of blood delivered to the cerebral circulation in a given time period, expressed in milliliters per minute. For the average adult, it is typically 750 mL/min, roughly 20% of cardiac output (CO) (Fantini et al., 2016). This equates to an average perfusion of 50 mL of blood per 100g of brain tissue. Factors affecting CBF include cerebral perfusion pressure (CPP) and total cerebrovascular resistance (tCVR), resulting in the equation CBF = CPP/tCVR. CPP is the pressure gradient which drives blood flow into the brain, and it is the difference between mean arterial pressure (MAP) and intracranial pressure (ICP). tCVR describes the resistance within the cerebrovascular bed which must be overcome to permit blood flow; factors affecting tCVR include those attributed to Poiseuille’s law, stating that blood flow (Q) through a vessel with length *L* and radius *r* driven by a pressure difference *∂P* is given by the equation:

$$Q=\pi r^{4}P/8\eta L$$

It is important to note that blood flow in cerebral vessels does not strictly obey Poiseuille’s law, since the vasculature is compliant and flow is often non-laminar. However, the equation illustrates that flow is highly sensitive to changes in vessel diameter, which represents the principal physiological mechanism for adjusting cerebral blood flow to meet local metabolic demands (Fantini et al., 2016). After only 3 min of oxygen deprivation, neurons begin to undergo extensive and irreversible damage; brain death may occur after 5 min (Fugate et al., 2022). This highlights the importance of maintaining adequate CBF, and in turn the regulatory systems that achieve this.

### 2.2 Regulatory mechanisms of CBF

As outlined above, cerebral blood flow is highly sensitive to changes in vessel diameter. Regulation of regional CBF is therefore primarily achieved through dynamic adjustments of vascular lumen, mediated by vasodilation and vasoconstriction across different segments of the cerebral circulation. Under normal physiological conditions, the systems that are responsible for CBF regulation can be divided into four categories: autoregulation, chemoregulation, endothelium-dependent regulation, and neuronal regulation (Claassen et al., 2021).

Autoregulation mainly concerns the change in cerebral blood flow in response to changes in CPP, which is determined by MAP as previously mentioned. ICP is relatively low under normal physiological conditions, exhibiting only small fluctuations, so changes in MAP are usually the main determinant of CPP. In pathological states, however, such as traumatic brain injury, intracranial tumors, or stroke, sustained elevations in ICP can critically reduce CPP and compromise cerebral perfusion, making ICP a key factor in CBF regulation in these contexts. According to the basic principles of hemodynamics, autoregulation maintains stable cerebral blood flow by adjusting cerebrovascular resistance in response to changes in CPP, primarily through alterations in vessel diameter. For example, an increase in blood pressure will result in vasoconstriction via contraction of vascular smooth muscle to normalize the transmural wall tension.

Chemoregulation describes the reaction of cerebrovasculature to changes in partial pressures of CO_2_ (and therefore pH), as well as O_2_. This type of regulation is termed CVR, which is one of the main focuses of this review article and will be described in more detail in Section 3.

Endothelium-dependent regulation describes the ability of endothelial cells to exert vasodilatory effects in response to neural and local metabolic stimulation. They play a crucial role in another focus of this article, NVC, which will be described in more detail in Section 3.

Finally, neuronal regulation describes two components of the involvement of neurons in CBF regulation; intrinsic parenchymal activation which essentially describes NCV, and extrinsic perivascular innervation of extraparenchymal vasculature (Claassen et al., 2021).

### 2.3 Structural differences across the cerebral vascular tree and their impact on regulatory mechanisms

**FIGURE 1:**
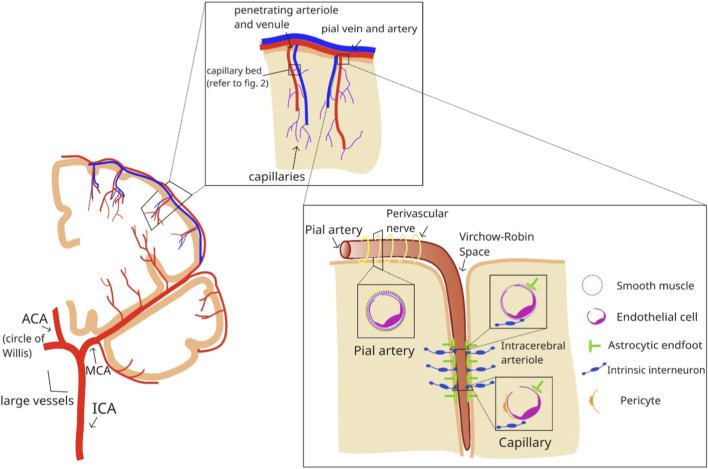
Schematic diagram demonstrating cerebrovasculature and histological changes seen across different vessel types: gross cross-sectional demonstration of larger vessels within circle of Willis, further progressing to focus on pail vessels, and finally focusing on smaller intracerebral vessels.

The structure of the cerebral vasculature changes systematically from large extraparenchymal arteries to capillaries, and these transitions are associated with shifts in the dominance of regulatory mechanisms (Figure 1). Progressive changes in vessel wall composition, including thinning of the tunica media, loss of elastic components, and increasing involvement of perivascular cells, alter the relative contribution of systemic and local regulatory pathways. In general, larger vessels are governed predominantly by systemic autoregulatory mechanisms, whereas smaller vessels are increasingly influenced by local metabolic and neuronal control, with CVR and NVC becoming dominant as vessel size decreases (Tan et al., 2013).

Blood is supplied to the brain via two main arterial systems: the carotid circulation, where the internal carotid artery contributes to the circle of Willis through the middle cerebral artery, and the vertebrobasilar system, in which the vertebral arteries join to form the basilar artery before giving rise to the posterior cerebral arteries. These pathways supply a hierarchical vascular tree in which structural specializations at different levels determine how blood flow is regulated.

Large extraparenchymal arteries, including the vessels of the circle of Willis and major pial arteries, possess a well-developed tunica media with multiple layers of smooth muscle (ranging from ∼20 layers in the internal carotid artery to 2–3 layers in smaller pial arteries) and a prominent internal elastic lamina (Cipolla, 2009). These features enable strong myogenic responses and pressure-dependent autoregulation. For example, increases in blood pressure are sensed through mechanical stretch, resulting in smooth muscle contraction and vasoconstriction (Claassen et al., 2021).

Penetrating arterioles, which branch from pial vessels within the Virchow-Robin space, exhibit fewer layers of smooth muscle and reduced elastic structures, marking the transition toward greater local control (Cipolla, 2009)While still responsive to autoregulatory mechanisms, they are more sensitive to local metabolites such as CO_2_ and K^+^ and play a larger role in NVC and CVR. Arterioles, with a single well-defined smooth muscle layer, are even less influenced by systemic regulation and rely predominantly on local metabolic and neuronal signals (Agarwal and Carare, 2021).

**FIGURE 2:**
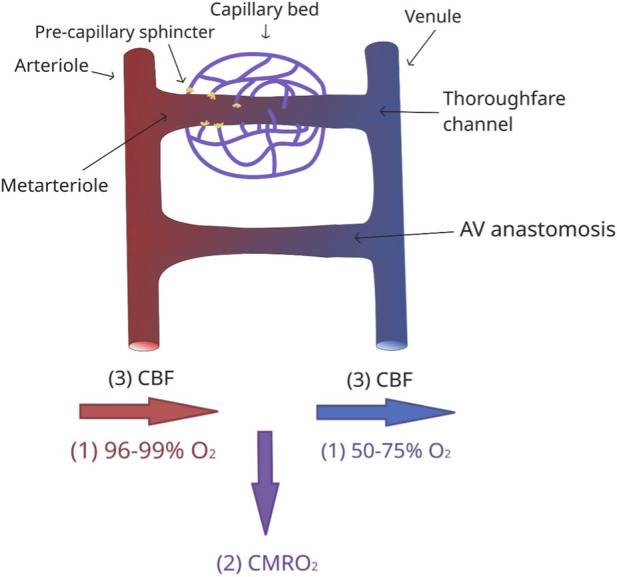
Simplified schematic diagram demonstrating closed pre-capillary sphincters and arteriovenous anastomosis. The thoroughfare channel connects the metarteriole to a venule. The direct connection between arteriole and venule is represented by the arteriovenous anastomosis. Below, three principal physiological parameters involved in cerebral energy homeostasis are shown: (i) arterial and venous oxygenation, (ii) cerebral metabolic rate of oxygen extraction (CMRO_2_), and (iii) CBF.

Precapillary sphincters, located at the transition between arterioles and capillaries, represent key sites of fine regulation. They consist of smooth muscle that dilates or constricts in response to local metabolic conditions, for example relaxing in the presence of increased CO_2_ or K^+^, thereby directing blood flow to active regions. A post-stimulus undershoot typically occurs 20–30 s after initial dilation to prevent hyperperfusion and edema due to elevated intravascular hydrostatic pressure (Grubb et al., 2020). When these sphincters close, blood can be diverted through preferential vascular pathways that effectively shunt flow from arterioles to postcapillary venules, thereby bypassing the capillary bed (Figure 2).

Capillaries, composed of a single endothelial layer with a basal lamina and lacking smooth muscle, are structurally specialized for exchange. Despite the absence of vascular smooth muscle, blood flow regulation persists at this level through the actions of pericytes (see Section 3.1) and endothelial cells, which play critical roles in NVC and CVR (Cipolla, 2009; Longden et al., 2017).

## 3 Neurovascular coupling and cerebrovascular reactivity

### 3.1 The neurovascular unit

**FIGURE 3:**
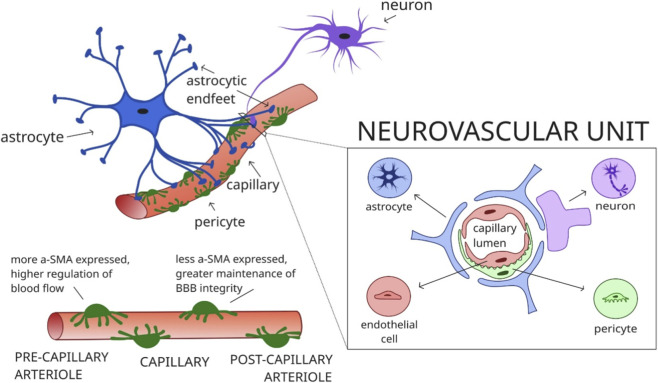
Schematic diagram demonstrating anatomical placement of astrocytic endfeet, cross-section of the neurovascular unit, and heterogenous a-SMA expression in pericytes.

The neurovascular unit (NVU) represents the structural and functional interface through which neuronal activity is coupled to local changes in blood flow. It comprises neurons, astrocytes, endothelial cells of the blood–brain barrier (BBB), pericytes, and extracellular matrix (ECM) components, which together coordinate rapid adjustments of vascular tone in response to changing metabolic demands (Guo et al., 2018). This functional interplay occurs primarily at the level of capillaries and precapillaries, where astrocytic endfeet and pericytes are in close contact with endothelial cells, forming the anatomical substrate for NVC (Figure 3).

Astrocytes are key intermediaries linking neuronal signaling to vascular responses. Their endfeet ensheath nearly the entire capillary surface, allowing for spatially restricted release of vasoactive molecules toward endothelial cells and pericytes (Figure 3). Their characteristic glial fibrillary acidic protein (GFAP) positive cytoskeleton enables this polarized organization. Astrocytes also communicate via gap junctions, allowing the propagation of Ca^2+^ waves across networks and coordinating responses to neuronal activation (Wei et al., 2025). When neurons become active, they release neurotransmitters and other mediators that stimulate astrocytes, triggering delayed intracellular Ca^2+^ elevations that lead to the localized release of vasoactive substances, including prostaglandins, nitric oxide (NO), and K^+^ ions, thereby mediating functional hyperemia (MacVicar and Newman, 2015; Staehr et al., 2020).

Endothelial cells rapidly respond to astrocytic and neuronal signals, releasing vasoactive factors and regulating BBB permeability. Key mediators include NO, prostaglandins (particularly PGE_2_), vascular endothelial growth factors (VEGF), and matrix metalloproteinases (MMPs) (Chen et al., 2014). These molecules act on vascular smooth muscle, pericytes, and endothelial targets to modulate vascular tone and barrier properties.

Pericytes are specialized mural cells surrounding capillaries that play a critical role in microvascular regulation, although their contractile capacity remains controversial due to conflicting experimental evidence (Zhu et al., 2022). Kisler et al. (2017) demonstrated reduced hemodynamic responses to stimulation in pericyte-deficient mice, supporting their active role. Hill et al. (2015) reported the absence of α-smooth muscle actin (aSMA) expression, suggesting limited contractility, but subsequent work showed heterogeneous aSMA expression across pericyte populations (Park et al., 2016). This discrepancy may stem from the rapid post-mortem depolymerization of actin (Alarcon-Martinez et al., 2018). Grant et al. (2019) identified transitional “ensheathing” pericytes at the precapillary–capillary interface, which express higher levels of aSMA than distal capillary pericytes, indicating functional heterogeneity along the vascular tree. Zambach et al. (2021) further demonstrated that aSMA expression correlates with contractile responsiveness and pressure distribution, underscoring the contribution of pericytes and precapillary sphincters to microvascular tone regulation (Figure 3) (Hartmann et al., 2021a; Chen et al., 2024; Attwell et al., 2016)^.^ Furthermore, while pericytes with minimal aSMA expression have been suggested to contribute more to BBB integrity rather than blood flow regulation, Isaacs et al. (2024) reported that they play a central role in sensing neuronal activity, signaling upstream to contractile pericytes and arterioles. This signaling is K^+^ dependent, activating a rapid propagation of retrograde hyperpolarization resulting in upstream arteriolar dilation (Longden et al., 2017).

Pericyte dysfunction, on the other hand, may result in critical consequences in the setting of various clinically relevant pathologies discussed in Section 5. One notable example is their possible contribution to the no-reflow phenomenon seen following endovascular thrombectomy reperfusion treatment after ischemic stroke, further explored in Section 5.4.

### 3.2 Defining the differences between NVC and CVR

Neurovascular coupling and cerebrovascular reactivity represent two distinct but complementary mechanisms regulating cerebral blood flow. NVC describes the activity-dependent regulation of microvascular tone, linking neuronal activation to local hemodynamic changes through coordinated signaling within the neurovascular unit. In contrast, CVR refers to the ability of cerebral vessels to change their diameter in response to alterations in arterial blood gas composition, particularly CO_2_ (PaCO_2_) (Phillips et al., 2016). Although these processes are most prominent at the level of capillaries and precapillaries, as described previously, they also influence upstream arterioles, albeit to a lesser degree.

**FIGURE 4:**
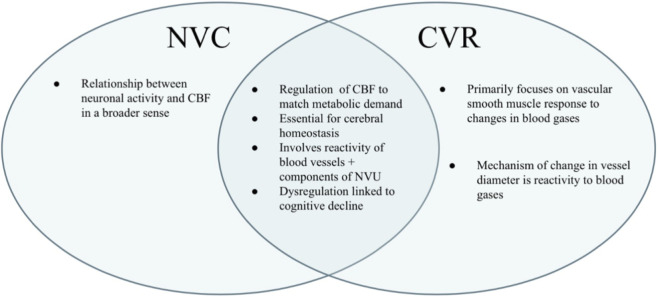
Venn diagram illustrating similarities and differences between NVC and CVR.

While NVC and CVR differ in their primary stimuli and mechanisms, neuronal activation versus blood gas changes, they share overlapping signaling pathways and cellular effectors. This is illustrated schematically in Figure 4, which demonstrates both the distinct and shared components of these two regulatory systems. This overlap likely contributes to the frequent interchangeable use of these terms in the literature, despite their mechanistic differences. NVC predominantly relies on neuron–astrocyte–endothelial signaling to adjust microvascular tone, whereas CVR operates across multiple vascular segments, responding to systemic changes in PaCO_2_ and pH. Together, they ensure both the spatial specificity (NVC) and the systemic adaptability (CVR) of cerebral blood flow regulation.

### 3.3 Mechanisms of CVR

Cerebrovascular reactivity reflects the capacity of cerebral vessels to constrict or dilate in response to changes in arterial blood gas composition, particularly CO_2_. Increased neuronal activity raises metabolic demand, leading to enhanced oxidative phosphorylation, oxygen consumption, and CO_2_ production (Gordon, 2024). This rise in PaCO_2_ and the associated decrease in extracellular pH are the primary stimuli driving vasodilation. The underlying chemistry follows the carbonic anhydrase equilibrium:

$$\left[H_{2}O\right]+\left[{\text{CO}}_{2}\right]⇆\;\left[H_{2}{\text{CO}}_{3}\right]⇆\;\left[{\text{HCO}}_{3}^{–}\right]+\left[H^{+}\right]$$

A drop in pH promotes the release of nitric oxide from endothelial cells and increases extracellular potassium concentration, both of which are potent vasodilators (Moriyama et al., 2022).

NO-mediated vasodilation occurs through activation of endothelial nitric oxide synthase (eNOS), increasing cyclic guanosine monophosphate (cGMP) in vascular smooth muscle, which lowers intracellular Ca^2+^ and induces relaxation (Battisti‐Charbonney et al., 2011). K^+^ contributes to vasodilation via activation of inwardly rectifying K^+^ channels, leading to hyperpolarization of vascular smooth muscle cells (Li and Yang, 2023). Both mechanisms act on vascular smooth muscle, precapillary sphincters, and pericytes, with particularly strong effects at the level of precapillaries and capillaries, highlighting the key role of these microvascular segments in CVR (Attwell et al., 2016; Hall et al., 2014).

**FIGURE 5:**
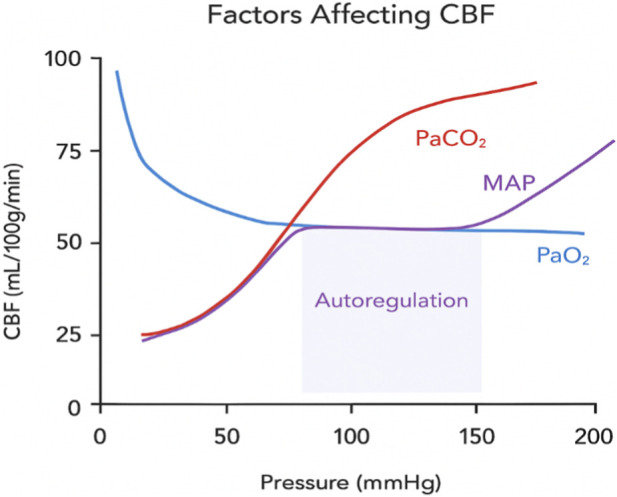
Schematic representation of factors affecting CBF.

Astrocytes also contribute to CVR through CO_2_-dependent intracellular Ca^2+^ signaling. Hypercapnia increases astrocytic Ca^2+^, which activates cyclooxygenase-1 (COX-1) and leads to PGE_2_ synthesis and release (Howarth et al., 2017). PGE_2_ acts on prostaglandin E2 receptor 4 (EP4) on endothelial cells, vascular smooth muscle cells, and pericytes, initiating vasodilatory signaling cascades (Zambach et al., 2021). This astrocyte-dependent pathway is modulated by glutathione availability, linking oxidative status to vascular responsiveness, and is impaired in pathological conditions such as stroke and schizophrenia (Howarth et al., 2017). Conversely, hypocapnia and elevated pH lead to vasoconstriction. Under alkaline conditions, altered amino acid ionization reduces eNOS activity and NO bioavailability (Battisti‐Charbonney et al., 2011). Decreased extracellular K^+^ and increased intracellular Ca^2+^ further promote vascular smooth muscle contraction. Figure 5 schematically illustrates the key factors influencing cerebral blood flow, summarizing the mechanisms discussed in this section with particular emphasis on the effects of changes in arterial CO_2_.

### 3.4 Mechanisms of NVC

Neurovascular coupling refers to the set of signaling mechanisms through which neuronal activity induces localized increases in cerebral blood flow. These mechanisms operate primarily at the level of capillaries and precapillaries, where neurons, astrocytes, pericytes, and endothelial cells of the neurovascular unit interact to regulate vascular tone (Rasband, 2016; Hosford and Gourine, 2019). Upon neuronal activation, the release of neurotransmitters, most notably glutamate, initiates intracellular signaling cascades in astrocytes and other NVU components, leading to the production of multiple vasoactive mediators that together generate functional hyperemia. This is supported by a study conducted by Staehr et al. (2025a), in which proteomics was used to identify key signaling molecules and pathways in astrocytes (including glutamatergic signaling), playing a key role in NVC.

Nitric oxide plays a central role in NVC. Glutamate released during synaptic activity stimulates neuronal nitric oxide synthase (nNOS) via NMDA receptor-mediated Ca^2+^ influx, resulting in rapid NO production. NO diffuses to nearby vascular smooth muscle cells and pericytes, activating guanylyl cyclase, increasing cGMP levels, and inducing vasodilation. NO also acts on endothelial cells, amplifying vasodilatory signaling (Hosford and Gourine, 2019).

Prostaglandins, generated through the COX pathway, represent another key signaling axis. Astrocytic activation leads to intracellular Ca^2+^ elevation, stimulating COX and resulting in the synthesis and release of vasodilatory prostaglandins, particularly PGE_2_. These act on EP4 receptors on vascular smooth muscle cells, endothelial cells, and pericytes to promote dilation (Hosford and Gourine, 2019).

K^+^ signaling constitutes a third important pathway. Increased neuronal activity elevates extracellular K^+^ concentration, activating inwardly rectifying K^+^ (Kir) channels on vascular smooth muscle cells (Li and Yang, 2023; Hosford and Gourine, 2019). This leads to hyperpolarization and relaxation of the vascular wall, thereby increasing local blood flow. Knockout of Kir2.1 subunits in mice reduces the NVC response by approximately 50%, highlighting the critical contribution of this pathway (Hosford and Gourine, 2019). Conversely, increased Kir2.1 expression is associated with an amplified NVC response, further supporting its role (Staehr et al., 2025b).

Astrocytic ATP and other gliotransmitters further modulate NVC (Beckers et al., 2024). ATP release can occur via Ca^2+^-dependent exocytosis (Sa et al., 2019) or through hemichannels (e.g., Cx43) (Iglesias et al., 2009) and pannexin channels, although the precise contribution of these mechanisms *in vivo* remains under investigation (Beckel et al., 2014). ATP acts on purinergic receptors on astrocytes, endothelial cells, and smooth muscle cells, propagating Ca^2+^ waves and triggering secondary release of NO and other vasoactive mediators (Coco et al., 2003; Wilson et al., 2022; Wang et al., 2023). A study using selective P2X7 blockade during induced seizures found significant reductions in vasodilatory responses, indicating that purinergic signaling contributes substantially during intense synaptic activation (Xu and Pelligrino, 2006; Pelligrino et al., 2011).

There is controversy in literature concerning which structures primarily dilate through NVC processes, as well as the timing and sequence of dilation. Some studies report that dilation occurs primarily in pial arteries and penetrating arterioles (Fernández-Klett et al., 2010). With regards to timing, some studies state that capillaries dilate before upstream arterioles (Fernández-Klett et al., 2010), with others reporting the opposite (Hartmann et al., 2021b). This discrepancy highlights a knowledge gap that is crucial to address in future studies, limiting the ability to gain a comprehensive understanding of this physiology.

These diverse pathways interact in a complex and partially redundant manner. A systematic review by Hosford and Gourine (2019) summarized *in vivo* studies employing pharmacological or genetic blockade of individual signaling pathways. Blockade of nNOS reduced the NVC response by an average of 64% across 11 studies, highlighting NO as the dominant mediator. Non-specific NOS blockade produced similar reductions. Inhibition of the COX pathway led to more than a 50% reduction in the NVC response, underscoring the importance of prostaglandins. Kir2.1 channel knockout resulted in a ∼50% reduction, demonstrating the significant contribution of K^+^ signaling. Together, these findings demonstrate that NVC relies on the coordinated action of multiple signaling cascades, ensuring robust and spatially precise regulation of cerebral blood flow.

## 4 Changes in neurovascular function across the lifespan: development, adulthood, and effects of aging

Neurovascular regulation evolves dynamically from embryonic development through aging, reflecting changes in cellular composition, vascular structure, and metabolic demands. Understanding these developmental trajectories is essential not only for interpreting experimental and imaging data across age groups but also for recognizing age- and development-related vulnerabilities relevant to clinical practice. It is important to note the lack of direct evidence demonstrating NVC and CVR development in early life, identifying a significant knowledge gap in the field and potential points of interest for future research.

### 4.1 General trends with development and early life

CBF undergoes marked developmental changes, reflecting evolving metabolic demands and structural maturation of the brain. Quantitative studies have shown that CBF peaks during early childhood and then declines progressively throughout life. Hirata et al. (2018) retrospectively analyzed quantitative rCBF using single-photon emission computed tomography (SPECT) in children aged 6 days to 178 months, showing a rapid increase during the first year, followed by a second acceleration between approximately 2–8 years, after which CBF gradually declines. These early years correspond to periods of intense cognitive, motor, and sensory development, associated with increased metabolic activity and maturation of NVC and CVR mechanisms.

The developmental trajectory of CBF is closely linked to three overlapping processes - synaptogenesis, myelination, and synaptic pruning, which occur at different rates across brain regions. Maturation follows an evolutionary sequence, with primary sensory areas (e.g., occipital lobe) maturing before associative and prefrontal regions (Taki et al., 2011). Synaptogenesis begins in mid-gestation, peaks around 2–3 years of age, and rises again during adolescence, particularly in the prefrontal cortex (Sar and nat, 2023; Wang et al., 2018). Myelination starts in late gestation and peaks during the first postnatal years, especially in primary sensory and motor cortices, supporting rapid white matter perfusion changes (Deoni et al., 2015; Miller et al., 2012). Synaptic pruning begins near birth, intensifies during childhood, and peaks between 10 and 20 years, refining neural circuits (Sager et al., 2020; Selemon, 2013).

**FIGURE 6:**
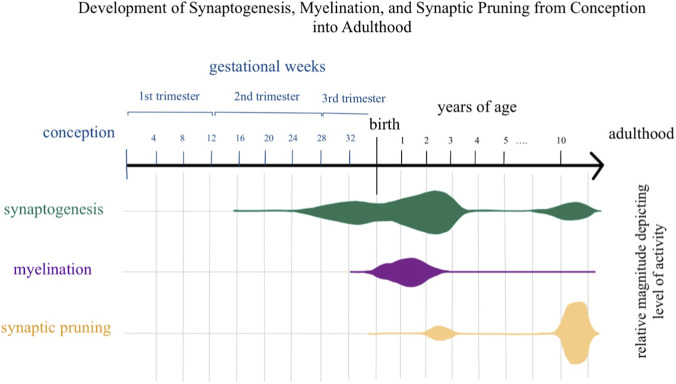
Simple schematic diagram visualizing synaptogenesis, myelination, and synaptic pruning processes from conception into adulthood. Horizontal graphical plots represent the activity of each process color coded, with an increase in size representing increased activity of the corresponding process.

These processes occur concurrently rather than sequentially, which explains why CBF remains elevated during early pruning phases: synaptogenesis and myelination continue to impose high energetic demands, necessitating sustained perfusion. Figure 6 summarizes the temporal dynamics of these processes, highlighting their overlap and their differential impact on gray and white matter vascular development.

### 4.2 Differences between white matter and grey matter development

Gray and white matter exhibit distinct developmental trajectories of CBF, reflecting differences in their cellular composition, metabolic activity, and vascular architecture. Gray matter contains a higher density of neurons and synapses, resulting in substantially higher metabolic demands and perfusion compared to white matter. Widely accepted average values in young adults illustrate this contrast: gray matter CBF is approximately 80 mL/100 g/min, whereas white matter CBF is about 20 mL/100 g/min (Fantini et al., 2016). During early childhood, both values are elevated, consistent with the global CBF peak described in the previous section.

An important parameter illustrating these differences is arterial transit time (ATT), defined as the time required for blood to travel from the arterial system to the capillary bed. ATT reflects microvascular structure and perfusion efficiency. In adults, ATT is generally longer in white matter than in gray matter due to lower capillary density and myelination-related flow dynamics. Interestingly, a recent pediatric ASL study reported the opposite pattern—longer ATT in gray matter—which was attributed to methodological factors including ROI selection, ASL standardization, and cerebellar confounds (Prysiazhniuk et al., 2025). When these factors were accounted for, white matter CBF showed a clear negative correlation with age, whereas gray matter CBF did not, reflecting the influence of increasing gray matter ATT during development (Prysiazhniuk et al., 2025). White matter regions also exhibited greater inter-individual variability in children, likely reflecting asynchronous axonal and vascular maturation. Together, these findings highlight that gray–white matter perfusion differences evolve dynamically with age and are sensitive to methodological choices, which is crucial for interpreting developmental NVC and CVR data.

### 4.3 General trends seen with aging

Aging is associated with widespread alterations in cerebral perfusion and metabolism, which directly affect NVC and CVR (Cai et al., 2023; Skøtt et al., 2025). As illustrated in Figure 2, the three principal physiological parameters involved in cerebral energy homeostasis are (i) arterial and venous oxygenation, (ii) cerebral metabolic rate of oxygen extraction (CMRO_2_), and (iii) CBF. CBF declines with age, with the prefrontal cortex, insular cortex, and caudate nucleus showing the most prominent reductions (Lu et al., 2011). These changes are heterogeneous across brain regions and reflect a combination of vascular remodeling, endothelial dysfunction, and altered metabolic demand.

As vascular compliance decreases and endothelial signaling becomes impaired, cerebrovascular resistance rises and vasodilatory capacity diminishes. To maintain adequate oxygen delivery, the brain compensates by increasing oxygen extraction, leading to lower global venous oxygenation and a higher oxygen extraction fraction. In parallel, declining nitric oxide bioavailability contributes to reduced endothelium-dependent vasodilation, further limiting cerebrovascular responsiveness (Beishon et al., 2021). The net effect is a progressive reduction in CVR, which represents one of the most robust physiological changes associated with aging at rest. These vascular and metabolic alterations disrupt NVC dynamics, leading to slower and attenuated hemodynamic responses to neuronal activity, which has important implications for both experimental interpretation and clinical evaluation in older individuals (Nowak-Flück et al., 2014).

### 4.4 Cerebrovascular remodeling

**FIGURE 7:**
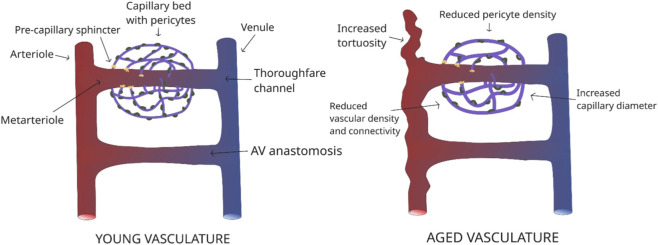
Schematic demonstrating cerebrovascular changes seen with aging.

Cerebrovascular remodeling represents a central mechanism underlying age-related changes in cerebral blood flow regulation. Bennett et al. (2024) investigated these alterations in aged mice, revealing increased vessel tortuosity, enlarged capillary diameters, and reduced vascular density and connectivity, as illustrated in Figure 7. These structural changes are accompanied by increased vessel stiffness, basement membrane thickening, and loss of vascular elasticity (Zimmerman et al., 2021), all of which impair the ability of the vasculature to adapt to fluctuating perfusion demands.

The increased stiffness primarily reflects altered ECM homeostasis, driven by increased collagen deposition, elastin degradation, and oxidative stress (Zimmerman et al., 2021; Balbi et al., 2015). With aging, oxidative stress activates the Smad pathway, promoting collagen synthesis and deposition, while upregulation of MMPs accelerates elastin breakdown (Balbi et al., 2015; Tucsek et al., 2014). Dysregulation of growth factor and cytokine signaling further contributes to ECM imbalance. As a result, the basement membrane thickens and vascular distensibility decreases, raising flow resistance and compromising microvascular perfusion (Zimmerman et al., 2021; Balbi et al., 2015).

Angiogenic capacity also declines with age, reducing capillary density and impairing vascular adaptability (Bennett et al., 2024; Balbi et al., 2015). Senescent cells accumulate in the aging vasculature, secreting pro-fibrotic and pro-inflammatory mediators such as TGF-β, IL-6, TNF-α, and MCP-1 (Balbi et al., 2015; Tucsek et al., 2014; Hu et al., 2025; Walek et al., 2023; Miller et al., 2019). This senescence-associated secretory phenotype reinforces ECM remodeling and inflammation, creating a feed-forward loop that exacerbates vascular dysfunction.

Together, these structural and molecular changes disrupt the dynamic properties of the vascular wall, diminishing both CVR and NVC. Increased vascular stiffness and reduced capillary density limit the speed and amplitude of hemodynamic responses to neuronal activity, while impaired ECM remodeling affects endothelial signaling and pericyte-endothelial interactions. These alterations contribute substantially to the blunted and delayed perfusion responses observed in aging, with implications for both basic research and clinical assessment of cerebrovascular health (Zimmerman et al., 2021; Balbi et al., 2015; Tucsek et al., 2014; Hu et al., 2025; Walek et al., 2023; Miller et al., 2019; Knox et al., 2022).

## 5 Neurovascular dysfunction in disease

Disruption of NVC and CVR plays a dual role in neurological disease - acting both as a driver of pathogenesis and as a consequence of ongoing pathology. Understanding these alterations provides crucial insight into disease mechanisms, clinical manifestations, and potential therapeutic strategies. This section summarizes how CBF dysregulation contributes to disease onset and progression and how different pathologies, in turn, impair these regulatory systems.

### 5.1 Neurodegenerative diseases

Neurodegenerative disorders are characterized by progressive neuronal dysfunction and loss, manifesting primarily as cognitive decline (e.g., Alzheimer’s disease [AD], vascular dementia) or motor impairment (e.g., Parkinson’s disease [PD], Huntington’s disease). The relationship between impaired CBF regulation and disease is bidirectional: altered NVC and CVR may contribute to disease development, while the disease processes themselves exacerbate vascular dysfunction. Common mechanisms include inflammation, oxidative stress, and BBB breakdown, which are more pronounced than in healthy aging (Knox et al., 2022; Chan et al., 2021).

BBB disruption is increasingly recognized as a key contributor to AD pathophysiology (Nation et al., 2019), including AD vasculopathy and inherited forms of cerebral amyloid angiopathy. Amyloid-β deposition in the extracellular matrix induces neuroinflammatory responses in cortical vessels, impairing vascular function. Astrocytic abnormalities, including swollen endfeet, loss of aquaporin-4 channels, and increased GFAP expression, further compromise NVC (Knox et al., 2022).

Vascular dementia, the second most common dementia after AD (Srinivasan et al., 2016), is a heterogeneous group of pathologies associated with chronic hypoperfusion, hypertension, atherosclerosis, or vasculitis. Prolonged ischemia initiates inflammatory cascades, generates reactive oxygen species, and leads to secondary CBF dysregulation, which accelerates cognitive decline. Conversely, BBB integrity is compromised by oxidative stress, pro-inflammatory signaling (MMP activation, cytokine release), and altered ECM metabolism, all of which impair NVC and CVR (Rosenberg, 2017; Andjelkovic et al., 2023).

Similar mechanisms are observed in PD, the second most common neurodegenerative disorder. Oxidative stress, MMP activation, and altered tight junction protein expression (e.g., decreased occludin) further weaken the BBB. Interestingly, deep brain stimulation has been shown to improve regional perfusion and motor function in PD, potentially through modulation of diseased microvasculature (Costea et al., 2019).

Overall, neurodegenerative diseases share convergent mechanisms that impair BBB integrity, increase oxidative and inflammatory stress, and disrupt NVC and CVR. These processes form self-reinforcing loops, whereby impaired regulation accelerates neurodegeneration, and ongoing degeneration further degrades cerebrovascular function.

### 5.2 Neuroinflammation

Neuroinflammation is a common feature of many CNS pathologies and affects both cerebrovascular reactivity (CVR) and neurovascular coupling (NVC). Activated microglia and perivascular macrophages release pro-inflammatory cytokines and chemokines that activate endothelial cells, upregulate adhesion molecules, and increase BBB permeability, leading to leukocyte infiltration and edema formation. These processes disrupt endothelial signaling and astrocyte–endothelial interactions, resulting in neurovascular uncoupling and reduced vasoreactivity (Smith et al., 2023). Consequently, the vasculature loses its ability to match perfusion to metabolic demand, amplifying tissue injury and disease progression.

### 5.3 Tumors

Alterations in CBF regulation are evident both within tumor tissue and in surrounding, non-tumorous brain regions. Several imaging studies have demonstrated reduced CVR ipsilateral to the tumor compared with the contralateral hemisphere, reflecting impaired vasoreactivity of the abnormal neovasculature characteristic of angiogenic tumors (Chow et al., 2016). Calvo-Imirizaldu et al. (2025) found a significant inverse correlation between tumor perfusion and CVR, consistent with structurally immature vessels that fail to respond appropriately to physiological stimuli. Importantly, Sebök et al. (2021a) reported that reduced CVR extends into peritumoral tissue, likely reflecting both occult tumor infiltration and the metabolic demands of the lesion affecting adjacent healthy tissue.

These alterations have direct clinical implications. Peritumoral hemodynamic dysfunction can affect functional MRI (fMRI) and CVR mapping, which are increasingly used for pre-surgical functional localization in eloquent cortical regions. Abnormal or blunted hemodynamic responses in these regions may lead to false-negative blood-oxygenation-level-dependent (BOLD) activations, complicating functional interpretation and surgical planning. Correcting or accounting for impaired CVR improves the reliability of functional mapping, which is critical for balancing maximal resection with preservation of neurological function.

### 5.4 Ischemic diseases

Severely decreased CBF disrupts cerebrovascular regulation both in acute and chronic conditions. In the acute ischemia, impaired perfusion leads to rapid endothelial dysfunction, pericyte loss, and breakdown of NVC and CVR. However, the detailed pathophysiology of acute cerebrovascular dysregulation in stroke lies beyond the scope of this review.

Chronic ischemization in cerebrovascular disorders is characterized by persistent reactive gliosis, oxidative stress, and structural remodeling, including glial scar formation and ECM alterations (Huang et al., 2014). These processes lead to long-term impairment of NVC and CVR, contributing to functional deficits. Importantly, altered vascular reactivity can be detected using functional and vascular imaging and provides diagnostic and prognostic information complementary to standard perfusion measures (Chan et al., 2021; Sunil et al., 2023). It has been shown that the degree of post-ischemic CVR reduction correlates with stroke severity and clinical outcome (Sim et al., 2016).

Regarding pericytes, their role in stroke pathophysiology is becoming increasingly understood, although there are controversies in literature. For example, following endovascular thrombectomy reperfusion treatment after ischemic stroke, the no-reflow phenomenon may occur, in which microvascular flow is absent or incomplete despite successful recanalization. This phenomenon has been attributed to residual thrombi, with recent research also suggesting pericyte dysfunction as a contributing factor. Further understanding the role of pericytes in both physiological conditions and disease is crucial, potentially recognizing promising therapeutic targets (Savitz et al., 2025; Shrouder et al., 2024).

Maximizing clinical response of recanalization procedures after ischemic stroke is an area of ongoing research, utilizing techniques to increase CBF before and after therapeutic interventions. This is known as cerebroprotection, in which strategies can be implemented before reperfusion, immediately after reperfusion, and in the recovery stages. For example, it has been shown that inhibiting glutamate excitotoxic signaling via administration of Nerinetide increased likelihood of better outcome in patients after reperfusion following ischemic stroke, likely due to the preservation of penumbral tissue. Additionally, strategies to dissolve residual thrombi following endovascular thrombectomy using alteplase or Tenecteplase have been shown to be safe and effective, possibly countering the no-reflow phenomenon seen in approximately one-third of patients. Finally, post-reperfusion therapy using antioxidants/anti-inflammatory agents have been investigated in clinical trials with mixed outcomes, indicating a need for further research in this area to better understand cerebroprotection targets in this stage (Savitz et al., 2025; Wechsler et al., 2023).

### 5.5 Psychiatric disorders

Psychiatric disorders such as depression, anxiety, and schizophrenia are associated with complex interactions between neural and vascular factors. The relationship between cerebrovascular dysfunction and psychiatric disease is bidirectional. On one hand, the vascular depression hypothesis proposes that cerebrovascular damage can impair mood-regulating networks, thereby contributing to the onset of depressive disorders (van Sloten et al., 2015; Huang et al., 2024). Conversely, chronic stress and depressive states induce neuroendocrine and inflammatory changes that secondarily impair vascular function, through alteration of potassium channel expression in cerebrovascular smooth muscle cells, resulting in impaired NVC (Burrage et al., 2018; Longden et al., 2014; Staehr et al., 2022).

Chronic stress activates the autonomic nervous system and hypothalamic–pituitary–adrenal axis, increasing catecholamines and cortisol. Persistent elevation of cortisol decreases nitric oxide bioavailability, both directly by inhibiting NOS (Liu et al., 2009) and indirectly by promoting oxidative stress (Iuchi et al., 2003), and reduces cAMP expression, impairing vasodilatory signaling. Pro-inflammatory mediator expression promotes arterial stiffness and vascular remodeling, leading to long-term declines in CVR and NVC. Given that approximately 35% of individuals worldwide report chronic stress (Smith and Wesselbaum, 2025), these mechanisms may have broad population-level implications for cerebrovascular and cognitive health.

In schizophrenia, increased serum inflammatory markers correlate with disease activity, and oxidative stress is evident in both chronic and new-onset cases. These alterations are associated with impaired CBF autoregulation and dysregulation of CVR and NVC (Najjar et al., 2017). Targeting these vascular mechanisms may offer opportunities for adjunctive therapeutic strategies beyond conventional psychopharmacology.

Alterations in CVR and NVC in these disorders can be detected using functional and vascular imaging, offering potential as complementary diagnostic or prognostic markers, although their clinical implementation remains limited.

### 5.6 Effects of disruption in the development of NVC and CVR processes

The maturation of NVC and CVR during prenatal and early postnatal periods represents a critical window for establishing long-term cerebrovascular regulation. Disruption of these processes can lead to persistent alterations in NVU structure and function, increasing vulnerability to later cognitive and neuropsychiatric disorders. Early-life insults, including inflammation, hypoxia-ischemia, metabolic disturbances, or severe emotional stress—can alter vascular signaling pathways, impair endothelial and glial interactions, and disrupt microvascular development (Stanimirovic and Friedman, 2012).

Such early disturbances may predispose individuals to long-term cerebrovascular dysregulation, which can manifest as altered NVC and CVR measurable later in life. Children exposed to significant perinatal risk factors may benefit from targeted follow-up and, where feasible, non-invasive imaging of NVC and CVR (e.g., ASL, BOLD CO_2_, TCD) as part of early screening strategies to identify subclinical vascular dysfunction before overt disease onset.

## 6 Methods of investigation

Assessing NVC and CVR provides essential insight into the integrity of CBF regulation. These methods are increasingly used not only in research but also for diagnosis, prognostication, and monitoring treatment responses in neurological disorders. Their reliability depends on rigorous protocols and physiologically meaningful interpretation. Table 1 below briefly describes and summarizes various techniques, including linked studies for further reading.

**TABLE 1:** Outline and summary of available techniques used to assess NVC and CVR.

Method	Principle	Outcome and clinical use	Literature (references)
Arterial spin labelling (ASL) MRI (Clement et al., 2022; Wintermark et al., 2005)	Subtraction technique between a control image and a labelled image (using magnetically labelled water molecules)	Provides quantitative perfusion maps in physiological units without the need for contrast, making it well suited for longitudinal and pediatric studies, although it is sensitive to motion and has lower spatial resolution than PET	Burley et al. (2021), Sebök et al. (2021b)
BOLD fMRI (van der Voort et al., 2024; Burley et al., 2021; Sebök et al., 2021b)	Utilizes difference in magnetic properties between deoxyhemoglobin and oxygenated hemoglobin, in which their concentration changes according to regional activity and subsequent oxygen extraction from local capillaries	Indirectly assesses CBF through changes in deoxyhemoglobin, enabling task-based or physiological challenge studies with high spatial resolution. Combining hypercapnia enables the creation of quantitative CVR maps that reveal both global and regional vascular dysfunction. These protocols are now well established and reproducible across centers. Currently represents the most standardized and widely applicable approach	Baska et al. (2024), Kaechele and Chakko (2025), Khan et al. (2025)
Transcranial doppler (TCD) (Wintermark et al., 2005; Sebök et al., 2021b; Baska et al., 2024)	Use of low-frequency transducer emitting ultrasonic waves which reflect off RBCs in vessels	Real-time measurement of flow velocity in basal arteries, widely used at bedside. Can combine hypercapnic challenge to assess changes in flow velocity relative to end-tidal CO_2_, yielding a CVR index	Sebök et al. (2021b), Khan et al. (2025), Csipo et al. (2019)
SPECT (Wintermark et al., 2005)	Produces a 3D image using the distribution of tissue-uptake of an injected radiotracer, allowing for analysis of perfusion	Provides a snapshot of cerebral perfusion based on first-pass tracer extraction, widely used in epilepsy through ictal–interictal comparisons (e.g. SISCOM)	Sebök et al. (2021b)
PET (Wintermark et al., 2005; Kaechele and Chakko, 2025)	Injection of radiotracers (using ^15^O-labelled water), capturing produced gamma rays	Able to measure metabolic activity as well as a quantitative measure of CBF. This method is technically demanding and limited to specialized centers	Sebök et al. (2021b), Senée et al. (2025)
Functional near-infrared spectroscopy (Khan et al., 2025; Csipo et al., 2019)	Novel non-invasive optical technique that measures changes in oxygenated and deoxygenated hemoglobin ratio similar to fMRI	Although spatially less precise than fMRI, it is portable and well suited for pediatric applications. Its current use remains mainly in research, but it offers potential for future clinical expansion	Lind et al. (2025), Lynch et al. (2021)
Retinal NVC mirroring brain NVC (Senée et al., 2025)	Adaptive optics rolling slit ophthalmoscope utilizing an off-axis phase contrast approach with camera-based slit gating	Non-invasive, high-speed, and high-resolution imaging technique capturing arterial and venular walls, allowing for visualization of vascular dynamics relevant to investigating NVC. Recently introduced technique undergoing research	Mangraviti et al. (2020)
2-photon microscopy (Lind et al., 2025)	Based on the principle of fluorophore excitation, utilizing two photons of higher wavelength to excite the fluorophore and result in emission of light. This enables real-time visualization of microvessel dynamics in deep tissue	Provides a much higher resolution image due to superior tissue penetration. Currently limited to experimental models. Higher costs and requires careful selection of fluorescent dyes for optimal yields	Wårdell et al. (2024)
Laser speckle contrast imaging (LSCI) (Lynch et al., 2021; Mangraviti et al., 2020)	Utilization of laser light which creates a random interference pattern (speckles) when illuminating cortical tissue. The motion of scattering particles, in this case red blood cells, disrupts this pattern and causes blurring. Blood flow is measured by quantifying the spatial blurring of the speckle pattern (speckle contrast) over time	Non-invasive, high-resolution technique that can be combined with hypercapnic challenges to assess CVR. Quantitatively assesses CBF and can be applied to intra-operative monitoring during neurosurgery. Investigations concerning CVR and NVC limited to experimental models	Mauritzon et al. (2022), Daněk et al. (2022)
Laser doppler flowmetry (Wårdell et al., 2024; Mauritzon et al., 2022; Daněk et al., 2022; Brožíčková and Otáhal, 2013)	Utilizes laser light emitted from a fiber optical probe, in which backscattered light signals from moving red blood cells are captured by a photodetector. The signal is processed to produce a perfusion value corresponding to microcirculation	Real-time measurement of CBF in which its implementation in neurocritical care and neurosurgery have been shown to be efficient and useful, although further evaluation is needed to establish routine use of these devices	Wårdell et al. (2024); Mauritzon et al. (2022); Daněk et al. (2022); Brožíčková and Otáhal (2013)

## 7 Conclusion

Neurovascular coupling (NVC) and cerebrovascular reactivity (CVR) are fundamental mechanisms maintaining the dynamic balance between neuronal activity, metabolism, and blood flow. Their regulation depends on tightly coordinated interactions within the neurovascular unit, integrating neuronal, glial, endothelial, and perivascular elements. Across the lifespan, these systems undergo profound developmental and age-related remodeling that shapes cerebral perfusion and determines the brain’s vulnerability to disease. Alterations in NVC and CVR are increasingly recognized as both drivers and consequences of diverse neuropathological processes—from neurodegeneration and chronic ischemia to tumors and psychiatric disorders. Understanding their physiological and structural bases is therefore essential not only for interpreting experimental and clinical perfusion data but also for linking microvascular function with systemic and molecular mechanisms of brain health and disease.

## 8 Future perspectives

Ongoing technological advances in neuroimaging are transforming our ability to probe cerebral perfusion with unprecedented spatial and temporal resolution. The physiological understanding of NVC and CVR thus becomes increasingly critical for interpreting these data and for translating them into clinically meaningful biomarkers. Modern MRI techniques now allow seamless integration of perfusion mapping with diffusion, metabolic, and structural imaging, providing a multidimensional view of the neurovascular unit. This convergence opens the way toward detecting subtle alterations in neurovascular interactions that likely precede overt pathology across a broad spectrum of neurological diseases. A deeper insight into these regulatory mechanisms will be essential to exploit emerging imaging capabilities for early diagnostics and, ultimately, for the development of truly causal and precision-based therapeutic approaches targeting neurovascular dysfunction.
